# Numerical and Experimental Validation of Mixing Efficiency in Periodic Disturbance Mixers

**DOI:** 10.3390/mi12091102

**Published:** 2021-09-14

**Authors:** Rubén R. López, Luz-María Sánchez, Anas Alazzam, Julia V. Burnier, Ion Stiharu, Vahé Nerguizian

**Affiliations:** 1 Department of Electrical Engineering, École de Technologie Supérieure, 1100 Notre Dame West, Montreal, QC H3C 1K3, Canada; anas.alazzam@ku.ac.ae (A.A.); vahe.nerguizian@etsmtl.ca (V.N.); 2 Cancer Research Program, RI-MUHC, McGill University, 1001 Decarie Boulevard, Montreal, QC H4A 3J1, Canada; julia.burnier@mcgill.ca; 3 Department of Engineering, Universidad Autónoma de Querétaro, Cerro de las Campanas, Santiago de Querétaro 76010, Querétaro, Mexico; 4 Department of Mechanical Engineering, Khalifa University, Abu Dhabi 127788, United Arab Emirates; 5 Department of Mechanical and Industrial Engineering, Concordia University, 1515 Saint Catherine West, Montreal, QC H3G 1M8, Canada; ion.stiharu@concordia.ca

**Keywords:** microfluidics, micromixers, mixing efficiency, image processing, numerical model

## Abstract

The shape and dimensions of a micromixer are key elements in the mixing process. Accurately quantifying the mixing efficiency enables the evaluation of the performance of a micromixer and the selection of the most suitable one for specific applications. In this paper, two methods are investigated to evaluate the mixing efficiency: a numerical model and an experimental model with a software image processing technique. Using two methods to calculate the mixing efficiency, in addition to corroborating the results and increasing their reliability, creates various possible approaches that can be selected depending on the circumstances, resources, amount of data to be processed and processing time. Image processing is an easy-to-implement tool, is applicable to different programming languages, is flexible, and provides a quick response that allows the calculation of the mixing efficiency using a process of filtering of images and quantifying the intensity of the color, which is associated with the percentage of mixing. The results showed high similarity between the two methods, with a difference ranging between 0 and 6% in all the evaluated points.

## 1. Introduction

Micromixers represent one of the most versatile component used in microfluidic systems [1]; they are used in chemical [2,3,4] and medical applications, such as nanoparticle synthesis [5]. Microfluidics and micromixing techniques have the potential to dispense controlled flows in the scale of nanoliters, while conditions at the microscale level remain relatively constant, bringing substances into close contact [4]. Micromixer channels are within 100 to 500 μm.

Mixing is a phenomenon involving the transport of a diluted species to increase its homogeneity. Micromixers operate typically under a laminar flow regime; in them, viscous forces dominate over inertial forces. Mixing at the microscale is based on three basic principles: molecular diffusion, chaotic advection and Taylor dispersion [6,7]. Molecular diffusion is related to the Brownian motion of molecules from a region of high concentration to one of low concentration. Chaotic advection is a process under which the influence of a flow scalar parameter change induced by the Lagrange flow dynamics leads to chaotic response even at low velocities [8]. Finally, Taylor dispersion arises from a distributed velocity field, e.g., a Poiseuille flow.

Micromixers are classified depending on the source that induces flow disturbances in active and passive mixers [9]. Active mixers use external force sources to introduce a perturbation in the flow and accelerate mixing [10]. This includes electro-kinetic [11], electroosmosis [12], ultrasound [13], dielectrophoretic [14] forces, among others. They are typically more difficult to operate and prone to failure due to their multiple components, and in some cases, moving parts. By contrast, passive micromixers use only fluid flow pumping as well as fixed geometry and shapes to induce flow perturbations [15,16]. Passive micromixers are generally easier to operate compared with active micromixers and do not require additional external energy sources other than fluid pumping. This includes T- and Y-shape micromixers, parallel lamination micromixers, sequential lamination micromixers, and chaotic advection micromixers [14,17,18,19].

Passive micromixers with 3D configuration have been shown to improve molecular diffusion and mixing due to their microchannel structure, which induces chaotic advection for efficient mixing. Moreover, they can yield consistent mixing [20,21]. However, these types of micromixers might be affected by clogging due to their complex three-dimensional configurations [22], while they are more difficult to fabricate compared to two-dimensional microfluidic devices. Hence, alternative approaches that are easy to produce are needed. Recently, one 2.5D configuration of periodic disturbance mixer (PDM) was proved to be a suitable alternative for the mixing of two liquids in the millisecond time range. This micromixer has been shown to produce controlled-size liposomes with diameters ranging from 52 nm to 200 nm [23]. Moreover, further research on how geometrical and dimensional features affect the mixing process inside this type of micromixer should be considered. In passive micromixers, efficient mixing is based on the structure of the microchannels; in particular, in micromixers with obstacles, it was found that the mixing efficiency increased at the highest barriers [1,24,25,26]. Table 1 shows several examples of micromixers and their mixing efficiency: most of these used the numerical model to quantify the mixing efficiency. In this study, we experimentally investigated the influence of the aspect ratio (AR) of the cross-section of microfluidic channels on the mixing process for the PDM via numerical modeling.

The paper is organized as follows: Section 2 contains the main stages of the proposed methods and details of the experimental tests. Section 3 includes the results and discussion. Finally, in Section 4, the conclusions of the article and future work are discussed.

## 2. Materials and Methods

The microfluidic devices used in the experiments were fabricated using standard soft lithography. Mixing inside the microfluidic device was evaluated using both a numerical model and experimental microscopic images with software image processing techniques.

### 2.1. Periodic Disturbance Mixer Design and Fabrication

**Figure 1:**
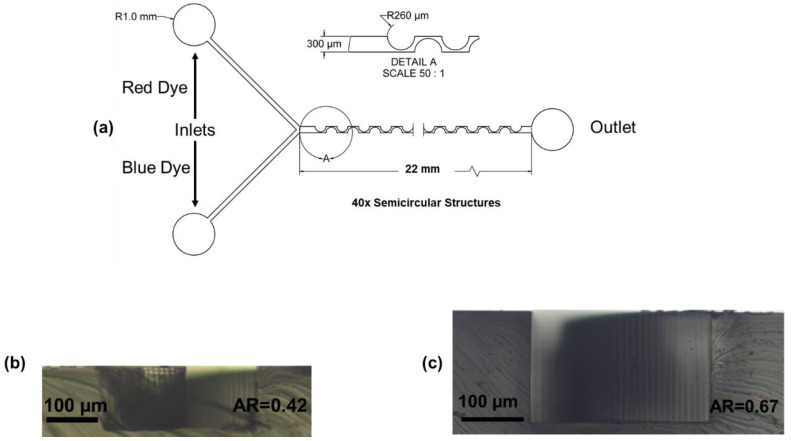
Periodic disturbance mixer: (**a**) schematics of the PDM; (**b**) microscopic image of the micromixer channel cross-section with an AR = 0.42, (**c**) AR = 0.67.

The micromixer has a mixing channel width of 300 μm and a variable height for different aspect ratios. The mixing channels consist of 40 semicircular structures with a radius of 260 μm placed on opposite directions along the mixing channel, as shown in Figure 1a. The same design was used for the numerical modeling of the mixing process using COMSOL Multiphysics 5.5. The data of the developed experiments are shown in Table 2.

The micromixer was fabricated using a standard soft lithography process. First, polydimethylsiloxane (PDMS) (Ellsworth Adhesives Canada, Stoney Creek, ON, Canada) was mixed with a curator agent in a 1:10 ratio. Then, it was poured onto a SU-8-produced negative mold. Bubbles in the mix were removed using a vacuum desiccator before and after pouring the contents onto the mold. The PDMS was cured for 4 h at 65 °C. The PDMS-produced microfluidic device was peeled from the SU-8 mold, then the inlets and outlets were pierced using a biopunch. The device was plasma-bonded onto a glass substrate (Globe Scientific Inc., Mahwah, NJ, USA). Tygon® Microbore and tubing (Cole-Palmer, Montreal, QC, Canada) were used to connect the device to 1 mL glass syringes (ILS Micro Syringes). The flow was controlled using two low-pressure neMESYS syringe pumps (Cetoni, Korbussen, Germany) controlled through a computer interface. Two main parameters were controlled: the total flow rate (TFR) measured in mL/h and the flow rate ratio (FRR), which is the ratio between the diluent and diluted species. In one inlet, water with red food dye was injected, while pure water was injected into another inlet. Two microfluidic devices were tested, one with an AR = 0.42, as shown in Figure 1b, and the second with an AR = 0.67, as shown in Figure 1c.

### 2.2. Numerical Modeling of Micromixing

The micromixing process was numerically modeled using the Navier–Stokes equations coupled with the convection–diffusion equation as a single-phase liquid (water). The next set of equations were numerically solved until a steady state was reached.

(1) $$\varrho \left(u\cdot ▽\right)u=▽\cdot [-pI+\mu \left(▽\mu +{\left(▽u\right)}^{T}\right)]+F$$

(2) ϱ▽·(u)=0

where ρ is the fluid density, *u* is the flow velocity, *p* is the pressure, μ is the dynamic viscosity, and *F* represents outer forces. The resulting velocity field from previous equations was used to numerically solve the convection–diffusion equations.

(3) ▽·(−D▽c)+u·▽c=R

(4) N=−D▽c+uc

where *c* is the diluted species concentration, *D* is the self-diffusion coefficient of water, *R* is the net volumetric source for the species, and *N* [Mol/m${}^{3}$] is the molar flux.

The mixing efficiency (ME) was calculated by considering a cross-section in the mixing channel perpendicular to the main flow direction. This cross-section was divided in a grid of 50 × 50 elements. The elements aligned with the height of the channel have a variable length. Then, the mixing efficiency (ME) was evaluated using the following formulation, based on the intensity of segregation [32,33].

(5) $$\mathrm{ME}=\left(1-\sqrt{\frac{\sigma^{2}}{\sigma_{0}^{2}}}\right)\times 100\%$$

where $\sigma_{0}^{2}$ is the variance of concentration at the beginning of the mixing channel (unmixed condition), and $\sigma^{2}$ is the variance of the concentration at a given cross-section [34]. The mixing efficiency was calculated using two methods, numerically with simulation and experimentally using image processing.

Table 3 shows the detailed information regarding the numerical model simulation [35]. Figure S1 shows the mesh independence for the mixing efficiency parameter.

### 2.3. Mixing Imaging

Experimental microscopic images were taken using an inverted microscope (Axio Observer, Carl Zeiss, Oberkochen, Germany) equipped with a high-speed camera (FASTCAM SA-X2-Full Frame-64G), that is, top-view images, perpendicular to the flow. The frame rate used was 1000 FPS with an exposure of 1 µs. These images were used for the quantitative evaluation of the mixing efficiency.

#### 2.3.1. Image Dataset of the Experimental Results

**Figure 2:**
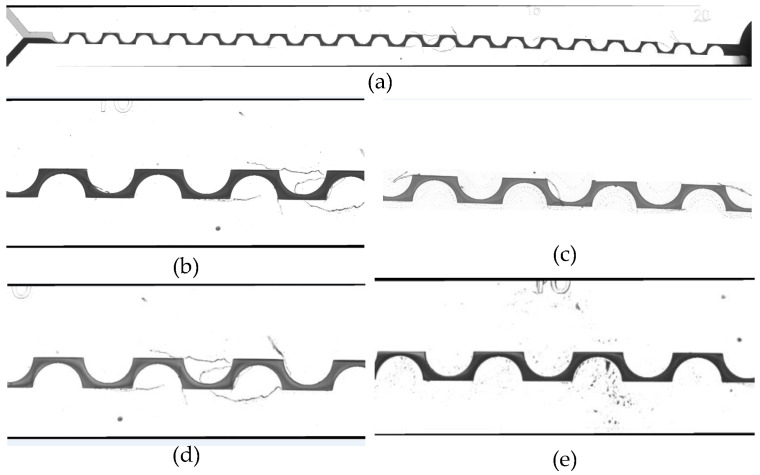
Examples of the processed images: (**a**) full stitched image; (**b**–**e**) fractions of processed images showing different noise levels and shades for the outline.

The database contains 26 images in .tif format, with dimensions of 14,940 × 1057 pixels and 96 dpi of resolution; they were obtained from the previously mentioned experimentation. The collection includes cases of images with different noise types and color intensity for the outline of the object of interest. The object of interest is the mixing channel, and the noise is the disturbances or variations in the image that prevent the determination of the corners and quantify the intensity of color in the corner fringes. Figure 2 shows examples of the images before processing: (a) the full image where it is observed that the black outline has a different thickness; (b–e) different noise types and disturbances that affect the mixing channel.

#### 2.3.2. Extraction and Evaluation of Characteristics

**Figure 3:**
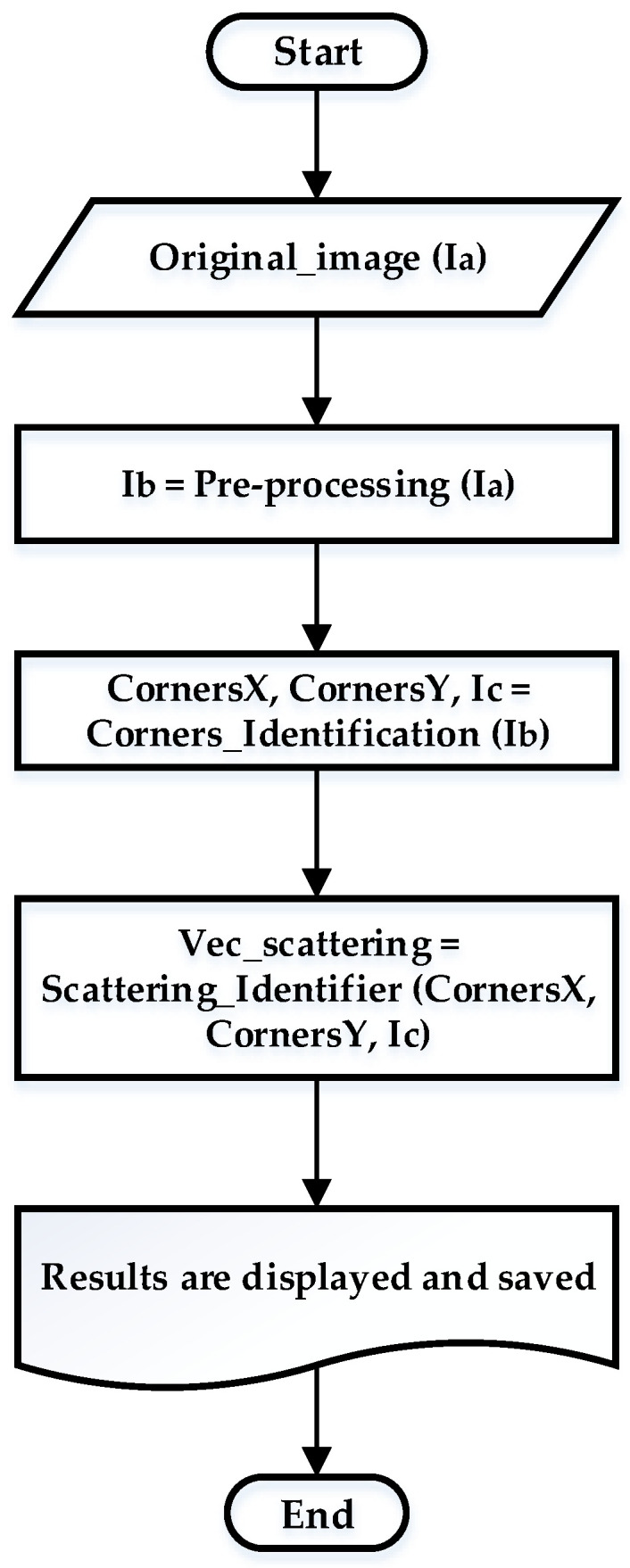
Flow chart of the proposed method for the automatic calculation of mixing efficiency.

**Figure 4:**
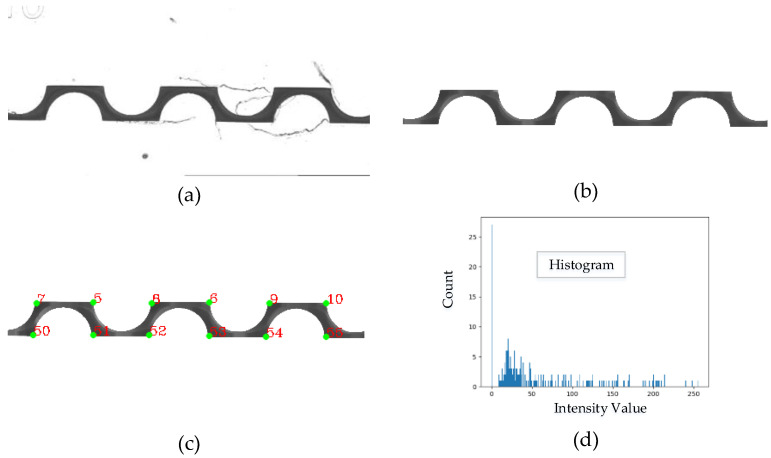
Steps of the image processing: (**a**) original image, (**b**) image without noise, (**c**) identification of corners, and (**d**) evaluation of color intensity.

The proposed method is based on the fundamental steps of digital image processing: image conditioning and identification of the object of interest (pre-processing), corner identification, evaluation of color intensity and efficiency calculation [36]. Figure 3 shows the flow chart diagram for the proposed method, and Figure 4 illustrates an example: (a) original image ($I_{a}$); (b) disturbances were removed from the mixing channel using a series of filters ($I_{b}$); (c) identification of the corners using the Harris method ($I_{c}$); (d) calculation of the histogram for each cross-section. The histogram shows the number of pixels, $n_{k}$, of each level of gray, $r_{k}$, which appears in the corner of interest. Each of the stages is described in the following subsections.

#### 2.3.3. Pre-Processing Stage

The process began by reviewing the size to confirm that all images had the same dimensions; then, the image was converted to grayscale, and the outline of interest was identified. The database contains several images with black edges, so they were removed to avoid interference at the calculation of the corners.

The process continued with the application of a Blurring filter, which can eliminate high-frequency content and noise. Subsequently, a morphological erosion was applied, followed by a morphological dilation, as this combination is useful in removing opening noise [15]. At the end of the second filtering stage, the noise was removed from the image, the edges were eliminated, and the channel contour was identified. Algorithm A1 (in the Appendix A) shows the pseudocode corresponding to the previous process, and the output of this stage is illustrated in Figure 4b.

#### 2.3.4. Identification of Corners and Evaluation of Gray Intensity

Before applying the method to find the corners, the Canny filter used to find the edges in the image was applied to make the corners stand out. The Harris method was used to identify the corners. The name of the method honors Harris’ discoverers and dates back to the 1980s. The technique is based on finding the intensity differences for a displacement of all directions [36].

**Figure 5:**
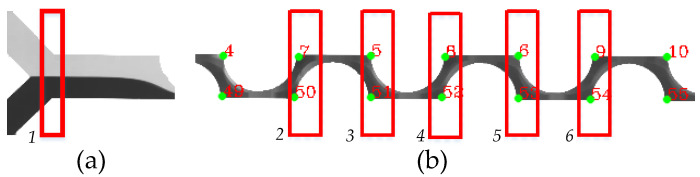
Cross-sections to calculate the efficiency: (**a**) reference section (r1); (**b**) sections that are selected for the efficiency calculation.

Once the corners were identified, then the coordinates were obtained, the data were stored in a matrix, and points were drawn in green on the image, Figure 5b. The coordinate matrix data and the contour were used to determine the fringes on which the histogram was to be evaluated. Afterwards, the coordinate matrix data were used to find all pairs of points that were in the same vertical location (different “y” coordinate and same coordinate “x”), which were identified as a cross-section. The histogram was used to determine the distribution of the intensities; in the case of the images, it shows value in pixels for each range of color intensity [36]. The results were stored in a vector and saved in a text file. Algorithm A2 (in the Appendix A) shows the pseudocode corresponding to this stage, and the output of this stage is illustrated in Figure 4c.

#### 2.3.5. Calculation of Mixing Efficiency

The information acquired from the color intensity vector was used for mixing efficiency calculations. The efficiency value was obtained as the division cross-sections of interest ($r_{2},r_{3},r_{4},\ldots ,r_{n}$) over the reference ($r_{1}$) and multiplied by one hundred to obtain the result as a percentage. Figure 5 shows the cross-sections used for the efficiency calculation, where section $r_{1}$ is considered as the reference.

#### 2.3.6. Training and Validation

The database was divided into two groups: 60% of the images were used for training and 40% for validation [37,38]. A rule-based classifier was used for training. The rules were defined from iterations varying the scales of the filters blurring, erosion and dilation until achieving the identification of the 32 cross-sections. The gray intensity along the channel was also sampled, and the basic concepts of flow and parameters of the experiment were applied. Once the information was collected, the literature of current methods for the quantification of mixing efficiency was reviewed, and Equation (6) was applied; gray quantification was obtained from the cross-section *n* between the gray quantification obtained at the beginning of the channel.

(6) $$\mathrm{ME}=\frac{GrayIntensity_{n}}{GrayIntensity_{1}}$$

The validation step was carried out using cross-validation, considering 5 repetitions; in each iteration, 60% were randomly selected for training and 40% for validation. The efficiency calculation results obtained by image processing from each iteration of the cross-validation was compared with those obtained by numerical models. In addition, to complete the validation stage, a color quantification sweep was made throughout the channel, and it was verified that at the end of the experiment, values between 95 and 100% were obtained for mixing efficiency. Table 4 shows three examples of the results obtained from gray quantification and their corresponding mixing efficiency values using Equation (6).

## 3. Results and Discussion

Previously, we reported a PDM device with an AR = 1 and a mixing time of 90% in the order of tens of milliseconds suitable for liposome production [39]. This micromixer uses both Taylor dispersion and Dean flow dynamics to enhance the mixing process. The changes in the cross area result in velocity magnitude changes, whereas the velocity vector direction is changed by alternatively shifting the semicircular structure center of the curvature, which induces centripetal forces that further enable the mixing process. The PDM curvilinear structure creates a periodical movement that transfers the diluted species in a perpendicular direction to the main liquid advection direction. This cyclic movement laminates the flow.

The height of the channel, hence the aspect ratio (AR), directly affects the mixing process: a device operated under the same flow conditions with smaller ARs will result in an increased velocity magnitude, Taylor dispersion and centripetal forces, which might reduce the mixing time according to simplified models [39,40]. In order to better understand the influence of the AR over the mixing process, two microfluidic devices with different AR were fabricated and their performances were evaluated. Two different methods were used to assess the mixing efficiency in the PDM: one using numerical modeling and the second using software image processing.

### 3.1. PDM Mixing at Different AR Numerical Modeling and Experimental Results

**Figure 6:**
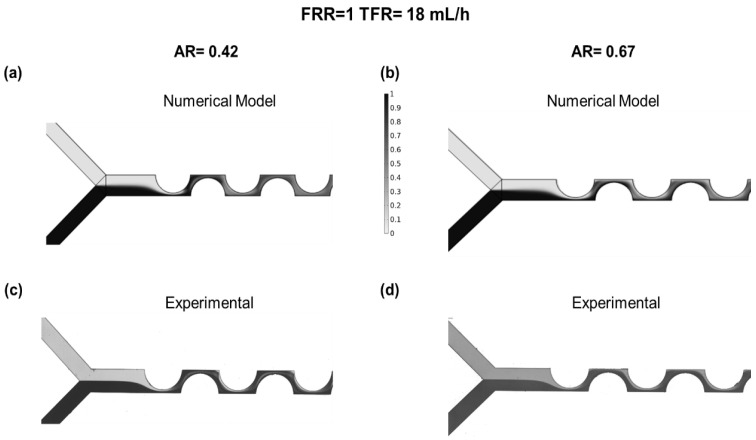
Mixing inside the microfluidic channels: (**a**) numerical model AR = 0.4164; (**b**) numerical model AR = 0.6733; (**c**) experimental AR = 0.42; (**d**) experimental AR = 0.67.

The mixing of the diluted species was analysed. From the numerical model, the mixing channel was divided into sections. At representative cross-sections, 2D images of the concentration profile were taken. The first section was located at the beginning of the mixing channel, and the second one was located immediately after the first curvilinear path, while the third one was located after the second one. The flow conditions in the numerically modeled device were set to FRR = 1 and TFR = 18 mL/h at different ARs (Figure 6). In Section 2 after the curvilinear path, the result of the centripetal forces is observed in the diluted species. In Section 3, the liquid is pushed in the opposite direction.

### 3.2. PDM Mixing Efficiency Performance at the Cross-Sections

**Figure 7:**
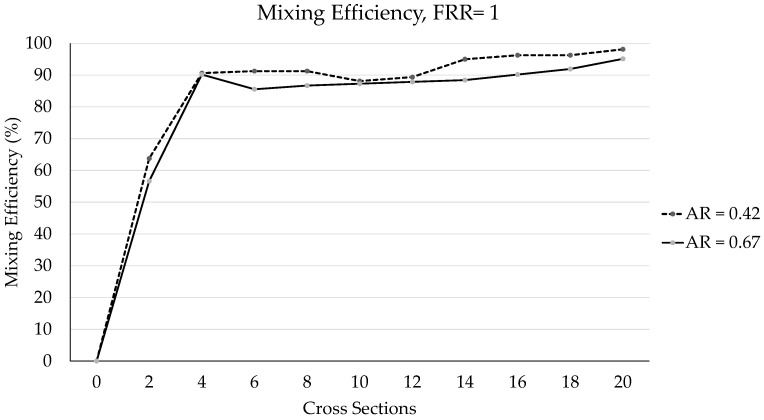
Mixing efficiency using the cross-sections shown in Figure 5 (r2, r4, r6, r8, …): TFR = 18 mL/h, FRR = 1.

The results of mixing efficiency using image processing for different ARs are shown in Figure 7. On the vertical axis, we have the efficiency percentage, and on the horizontal axis, the cross-sections. According to the results, the percentage of mixing increased as the cross-sections progressed; in the last sections, there are values between 95 and 100%. The behavior for both ratios is very similar, mainly in the first and last points of the graph; the greatest difference found is in graph point 14 with a difference of 5%. The cross-sections plotted correspond to the points identified in Figure 5. The experiment was repeated for two different ARs, and six different FRR values.

**Figure 8:**
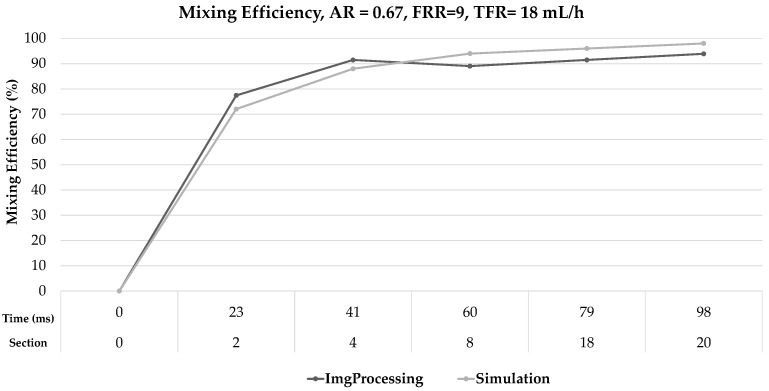
Comparison between simulation results and those obtained in image processing: AR = 0.67, FRR = 9; TFR = 18 mL/h.

**Figure 9:**
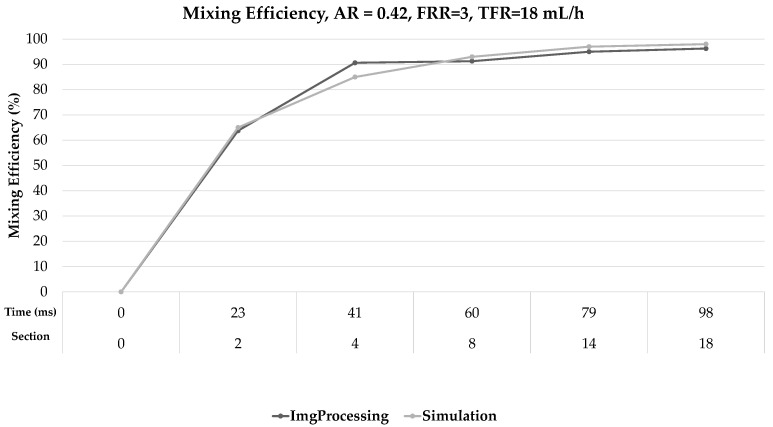
Comparison between simulation results and those obtained in image processing: AR = 0.42, FRR = 3; TFR = 18 mL/h.

Figure 8 shows the results of the comparison between the mixing efficiency data obtained from the numerical model and the experimental data (using image processing) for AR = 0.67 and FRR = 9. The maximum difference for this case was 6% at 41 ms, and the minimum difference was 1% at 51 ms and 79 ms. Figure 9 shows the results of the comparison between the mixing efficiency data for a different AR and FRR; in this case, the maximum difference was 5% at 60 ms, and the minimum was 0% at 32 and 51 ms. Considering all the cases described in Table 2, the maximum difference was 6% for FRR = 1, AR = 0.67, and the minimum difference was 0% for FRR = 3, AR = 0.42.

## 4. Conclusions

In this work, we performed the implementation, comparison and evaluation of two methods to calculate the mixing efficiency—the quantification of mixing using a numerical model and image processing. It was shown that the results in both methods are very close, achieving a difference between 0 and 6% in any of the cross-sections.

The microscopy-based mixing validation is a feasible method for evaluating the efficiency of microfluidic mixers: both Comsol modeling with experimental imaging and processing are efficient analysis methods, and depending on the conditions, time and development equipment (camera or software tools), we could apply either approach with the certainty of achieving reliable results.

The method based on image processing proved to achieve a fast response, and to execute the whole calculation process for the mixing efficiency automatically, it is sufficient to have the full channel image. The software removes noise, identifies corners to define cross-sections, quantifies gray intensity, and associates the intensity result with mixing efficiency. In addition, during the design of the algorithm, it was considered to design a user-friendly interface, in a free access programming language and modular architecture. The development of this method contributes to creating and improving robust tools for the evaluation of micromixers.

According to the results obtained from the calculation of mixing efficiency using the two methods, the PDM microfluidic efficiency showed higher mixing levels at a lower AR for all the FRR and TFR used.

## Figures and Tables

**Table 1:** Micromixers based on curvilinear paths used for liposome production.

Micromixer	Demonstrated Production Rate	Mixing Efficiency	Reference
Contraction Expansion Array (CEA)	24 mL/h	90% (120 ms whole channel)	[27]
iLiNP	30 mL/h	80–90% (<10 ms)	[22]
Periodic disturbance mixer (PDM)	18 mL/h	90% (approx. 95 ms)	[23]
MiliReactor	600 mL/h	Not mentioned	[28]
Serpentine Micromixer	9 mL/h	Not mentioned	[29]
Toroidal mixer design (TrM)	20,000 mL/h	Not mentioned	[30]
Staggered Herringbone Micromixer	60 mL/h	>80%	[17]
Mixer utilizing sharp corner structures	12 mL/h	>35%	[31]

**Table 2:** Experiment data: The flow-through microfluidic channels.

Parameter	Value			
AR	**0.42**		**0.67**	
Re	68	19, 30, 41, 53, 64	47	13, 21, 28, 36, 44
TFR (mL/h)	18	5, 8, 11, 17	18	5, 8, 11, 17
FRR	1, 3, 5, 7, 9, 12	8.56	1, 3, 5, 7, 9, 12	8.56

**Table 3:** Numerical model detailed information.

Property	Value
Mesh Vertices	1,566,514
Number of Elements	5,685,617
Minimum Element Quality	0.01205
Average Element Quality	0.6294
Element Volume Ratio	$1.95\times 10^{-4}$
Mesh volume m${}^{3}$	$6.28\times 10^{-10}$

**Table 4:** Results of calculating gray intensity, mixing efficiency using image processing and the numerical model (AR = 0.42, TFR = 18 mL/h, Table S1).

Data	Time (ms)	0	23	32	41	51	60	79	Final
FRR = 1	Gray intensity	169	98	101	131	139	151	154	161
	% ME − Img Processing	0%	58%	60%	78%	82%	89%	91%	95%
	% ME − Numerical Model	0%	55%	57%	77%	79%	89%	94%	97%
FRR = 3	Gray intensity	169	102	115	146	146	147	158	162
	% ME − Img Processing	0%	61%	68%	86%	86%	88%	93%	95%
	% ME − Numerical Model	0%	65%	68%	85%	86%	93%	97%	98%
FRR = 9	Gray intensity	169	133	153	148	158	161	166	161
	% ME − Img Processing	0%	78%	90%	87%	93%	95%	98%	95%
	% ME − Numerical Model	0%	81%	85%	93%	94%	97%	99%	99%

## Data Availability

The information is added in Supplementary Information.

## Supplementary Materials

The following are available online at https://www.mdpi.com/article/10.3390/mi12091102/s1, Figure S1: Mixing Efficiency vs Number of Mesh Elements for FRR = 1, TFR = 18 mL/h at AR = 0.42 at 23 ms from the beginning of the channel, Table S1: Results of calculating gray intensity, mixing efficiency using image processing and the numerical model (TFR = 18 mL/h).

## Appendix A

Image processing stage pseudocodes to calculate mixing efficiency.

**Algorithm A1**. Proposed method to remove noise, showing lines 1–9, corresponding to the images in Figure 3b.
**Input:** $I_{a}$, input image.
**Output:** $I_{f}$, denoised image, identified contour.
1: **procedure** Pre-processing ($I_{a}$)
2: $height,width=getShape(I_{a})$
3: $I_{b}=convertRGBtoHSV\left(I_{a}\right)$
4: $I_{c}=getContours\left(I_{b}\right)$
5: $I_{d}=croppedImg\left(I_{c},scale*width,scale*high\right)$ ▹ scale is thickness
6: $I_{e}=filterBlur\left(I_{d}\right)$
7: $I_{f}=morphologyErosion\left(I_{e},K_{1}\right)$
8: $I_{f}=morphologyDilation\left(I_{f},K_{2}\right)$ ▹ K1, K2 are the kernel
9: $return(I_{f})$

**Algorithm A2**. Proposed method for identifying corners, showing lines 1–11, corresponding to the images in Figure 3d.
**Input:** $I_{a}$, input image.
**Output:** $I_{d}$, identified corners, CornersX, CornersY.
1: **procedure** IntensityEvaluation($I_{a}$)
2: thickness=4 ▹ thickness of stripes in pixels
3: $I_{b}=convertRGBtoGRAY\left(I_{a}\right)$
4: $I_{c}=filterCanny\left(I_{b}\right)$
5: $Corners_{X},Corners_{Y},I_{d}=getCorners\left(I_{c}\right)$
6: **for all** *i* **do**
7: $I_{d}=cornersImg\left(I_{a},thickness,Corners_{X}\left[i\right],Corners_{Y}\left[i\right]\right)$
8: $HistMask=getHist(I_{d})$
9: Scattering=Sum(HistMask)
10: VectScattering[i]=scattering

11: $return(Corners_{X},Corners_{Y},I_{d})$
