# Supplementary material for: Numerical and Experimental Validation of Mixing Efficiency in Periodic Disturbance Mixers
Source: Micromachines (Basel). 2021 Sep 14;12(9):1102. doi: 10.3390/mi12091102 (PMC8469598; doi:10.3390/mi12091102)
Supplement: Supplementary file 1 [file micromachines-12-01102-s001.zip › micromachines-1364010-supplementary.pdf]

Supplementary Information

Numerical and Experimental Validation of Mixing Efficiency in Periodic Disturbance Mixers

Rubén R. López [1,2*], Luz-María Sánchez [3*], Anas Alazzam[4,1], Julia V. Burnier [2], Ion Stiharu[5] and Vahé Nerguizian[1]

[1] Department of Electrical Engineering, École de technologie supérieure, 1100 Notre Dame West, Montreal, Quebec, Canada; ruben.lopez-salazar.1@ens.etsmtl.ca,vahe.nerguizian@etsmtl.ca (V. N.)

[2] Cancer Research Program, RI-MUHC, McGill University, 1001 Decarie Boulevard, Montreal, Quebec H4A 3J1, Canada; julia.burnier@mcgill.ca (J.V.B.)

[3] Department of Engineering, Universidad Autónoma de Querétaro Cerro de las Campanas s/n, Santiago de Querétaro, Querétaro, México

[4] Department of Mechanical Engineering, Khalifa University, Abu Dhabi, UAE; anas.alazzam@ku.ac.ae (A.A.)

[5] Department of Mechanical and Industrial Engineering, Concordia University, 1515 Saint Catherine West, Montreal, Quebec, Canada; ion.stiharu@concordia.ca (I.S)

* These authors contributed equally to this work.

Correspondence: ruben.lopez-salazar.1@ens.etsmtl.ca (R.R.L.); luzmsr@ieee.org (L.-M.S.);
Tel.: +1-915-232-0336 (R.R.L.)

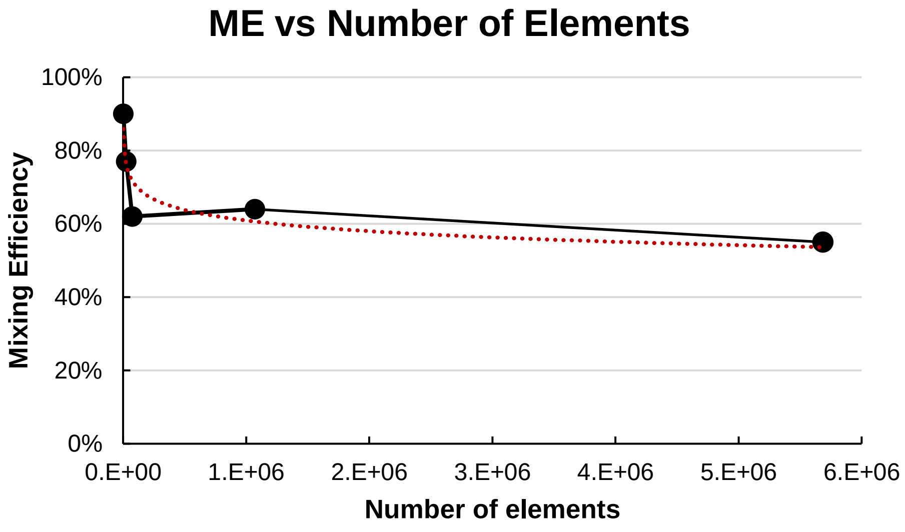

Figure S1. Mixing Efficiency vs Number of Mesh Elements for FRR=1, TFR=18 mL/h at AR=0.42 at 23 ms from the beginning of the channel. The red trendline shows an asymptotical behavior above 5 million of mesh elements.

Table S1. Results of calculating gray intensity, mixing efficiency using image processing and the numerical model (TFR = 18mL/h). These results correspond to the percentage of images used for the validation step, the first iteration of cross-validation.

**Data**	**Time (ms)**	**0**	**23**	**32**	**41**	**51**	**60**	**79**	**Final**
**FRR = 1 TFR = 18 mL/h AR = 0.42**	Gray intensity	169	98	101	131	139	151	154	161
	% ME - Image Processing	0%	58%	60%	78%	82%	89%	91%	95%
	% ME – Numerical Model	0%	55%	57%	77%	79%	89%	94%	97%
**FRR = 3 TFR = 18 mL/h AR = 0.42**	Gray intensity	169	102	115	146	146	147	158	162
	% ME - Image Processing	0%	61%	68%	86%	86%	88%	93%	95%
	% ME - Numerical Model	0%	65%	68%	85%	86%	93%	97%	98%
**FRR = 9 TFR = 18 mL/h AR= 0.42**	Gray intensity	169	133	153	148	158	161	166	161
	% ME - Image Processing	0%	78%	90%	87%	93%	95%	98%	95%
	% ME - Numerical Model	0%	81%	85%	93%	94%	97%	99%	99%
**FRR = 5 TFR = 18 mL/h AR= 0.42**	Gray intensity	167	118	122	144	152	157	159	163
	% ME - Image Processing	0%	71%	73%	86%	91%	94%	95%	98%
	% ME - Numerical Model	0%	68%	73%	87%	88%	94%	97%	99%
**FRR = 7 TFR = 18 mL/h AR= 0.42**	Gray intensity	165	119	130	141	154	157	159	161
	% ME - Image Processing	0%	72%	79%	85%	93%	95%	96%	98%
	% ME - Numerical Model	0%	76%	81%	91%	92%	96%	98%	99%
**FRR = 12 TFR = 18 mL/h AR= 0.42**	Gray intensity	165	120	132	147	154	157	159	163
	% ME - Image Processing	0%	73%	80%	89%	93%	95%	96%	99%
	% ME - Numerical Model	0%	77%	83%	92%	93%	97%	99%	99%
**FRR = 1 TFR = 18 mL/h AR= 0.67**	Gray intensity	164	53	59	77	89	102	122	139
	% ME - Image Processing	0%	32%	36%	47%	54%	62%	74%	85%
	% ME - Numerical Model	0%	29%	37%	47%	55%	63%	75%	83%
**FRR = 3**	Gray intensity	173	84	95	121	132	142	155	161

**TFR = 18 mL/h AR= 0.67**	% ME - Image Processing	0%	49%	55%	70%	76%	82%	90%	93%
	% ME - Numerical Model	0%	44%	55%	69%	73%	81%	88%	92%
**FRR = 5 TFR = 18 mL/h AR= 0.67**	Gray intensity	162	82	101	127	132	141	150	159
	% ME - Image Processing	0%	51%	62%	78%	81%	87%	93%	98%
	% ME - Numerical Model	0%	48%	61%	76%	79%	86%	91%	95%
**FRR = 7 TFR = 18 mL/h AR= 0.67**	Gray intensity	166	105	123	140	147	152	158	162
	% ME - Image Processing	0%	63%	74%	84%	89%	92%	95%	98%
	% ME - Numerical Model	0%	63%	73%	84%	86%	91%	95%	97%
**FRR = 9 TFR = 18 mL/h AR= 0.67**	Gray intensity	168	122	138	142	152	154	159	165
	% ME - Image Processing	0%	73%	82%	85%	90%	92%	95%	98%
	% ME - Numerical Model	0%	72%	79%	88%	90%	94%	96%	98%
